# Study on the correlation between resilience, social support and quality of life in patients with inflammatory bowel disease

**DOI:** 10.3389/fpsyt.2025.1694513

**Published:** 2026-01-22

**Authors:** Yanfei Yu, Yeping Zheng, Lingsha Wu

**Affiliations:** The Second Hospital of Jiaxing, Jiaxing, Zhejiang, China

**Keywords:** inflammatory bowel disease, mediating effect, quality of life, resilience, social support

## Abstract

**Background:**

Inflammatory bowel disease (IBD) seriously affects the physical and mental health of patients. Resilience can enhance patients’ coping abilities and improve their health to a certain extent, as well as their quality of life. Social support is closely related to quality of life. However, there is currently a lack of research on the relationship between resilience, social support and quality of life in patients with IBD.

**Objective:**

To explore the mediating role of social support between resilience and quality of life in patients with IBD.

**Methods:**

This study was a cross-sectional study. A total of 207 IBD patients from the Department of Gastrointestinal Surgery of a tertiary first-class hospital in Jiaxing City were selected by convenience sampling from August 2023 to April 2024. They were investigated using a general information questionnaire, Connor-davidson Scale (CD-RICS), Quality of Life Scale, and Social Support Rating Scale. The relationships among resilience, social support, and quality of life were analyzed. A total of 207 questionnaires were distributed, and 207 valid questionnaires were recovered.

**Results:**

The average age of 207 patients with inflammatory bowel disease was (44.24 ± 14.10) years old. The total resilience score of IBD patients was (61.58 ± 22.37) points, the total social support score was (43.37 ± 11.46) points, and the total quality of life score was (182.22 ± 31.94) points. The mediating effect analysis showed that social support played a partial mediating role between resilience and quality of life in IBD patients, accounting for 32.35% of the total effect.

**Conclusion:**

The quality of life of IBD patients is at a moderate to high level. Resilience in IBD patients can directly affect their quality of life, and also indirectly affect it through social support. Medical staff and society should take measures to improve the social support of IBD patients, thereby helping them live better lives.

## Highlights

- What is already known? There is a lack of research on the relationship among resilience, social support, and quality of life in IBD patients.
- What is new here? Resilience, social support and quality of life in patients with inflammatory bowel disease are closely related. In addition, social support partially mediates the relationship between resilience and quality of life.
- How can this study help patient care? It guides medical professionals and society to take steps to improve social support for people with IBD, thereby helping them to live better lives.

## 1 Introduction

Inflammatory bowel disease (IBD) is a group of chronic, non-specific inflammatory conditions of the gastrointestinal tract of unknown etiology, primarily comprising ulcerative colitis (UC) and Crohn’s disease (CD) (1, 2). Characteristic symptoms include abdominal pain, diarrhoea, and haematochezia, with a higher prevalence among young and middle-aged adults. The disease’s protracted course and high relapse rate cause significant disruption to patients’ studies, work, and daily lives, severely impairing their overall well-being and reducing their quality of life (QoL) (3).Beyond this clinical burden, the onset and progression of IBD are closely linked to psychosocial factors. Substantial evidence from systematic reviews and meta-analyses indicates that IBD patients frequently experience significant psychological and psychopathological features, such as affective disorders (anxiety and depression), somatisation, alexithymia, and specific defence mechanisms. These factors interact bidirectionally with disease activity, collectively contributing to the complex biopsychosocial model of IBD (4–8). Consequently, focusing on patients’ positive psychological resources is crucial for comprehensive disease management. Among these resources, resilience is particularly important. It refers to an individual’s capacity to recover or maintain relatively stable physiological and psychological functioning when confronted with major stressors such as adversity, trauma, threats, or emergencies (9). Theoretical models posit resilience as a key protective factor that enhances an individual’s ability to cope and adapt to diverse challenges, thereby significantly improving mental and physical health and promoting a better QoL (10– 11). Social support, another critical resource, denotes the degree of material and emotional connection an individual perceives from their social network. Research consistently shows a positive correlation between perceived social support and QoL in IBD patients (12, 13). Critically, a dynamic relationship exists between resilience and social support. Theoretical models, such as the Social Support Deterioration Model, suggest that social support fosters resilience, implying that greater social support leads to higher resilience levels (14). This proposed mediating mechanism finds support in clinical populations; for instance, resilience has been demonstrated to mediate the relationship between interpersonal risk factors and hopelessness in depression (15). Similarly, in patients with other chronic conditions like cancer, resilience acts as a key mediator influencing QoL (16).

Currently, research specifically examining the interrelationships between resilience, social support, and QoL in IBD patients remains limited. To address this gap, this study aims to investigate the mediating role of social support in the relationship between resilience and QoL in patients with IBD. We hypothesise that: 1. Resilience will directly and positively predict QoL in IBD patients. 2. Social support will partially mediate the relationship between resilience and QoL. By testing this mediation model, this research seeks to provide a theoretical foundation for developing psychosocial interventions aimed at enhancing the quality of life for individuals living with IBD.

## 2 Subjects and methods

### 2.1 Study subjects

IBD patients from the Department of Gastrointestinal Surgery of a tertiary first-class hospital in Jiaxing City were selected by convenience sampling from August 2023 to April 2024. Inclusion criteria: (1) Patients clearly diagnosed with IBD according to the Consensus Opinions on the Diagnosis and Treatment of Inflammatory Bowel Disease (Beijing, 2018) (17); (2) Aged ≥ 18 years; (3) Having certain reading and writing abilities, good communication skills, and being able to complete the questionnaire alone or with assistance; (4) Voluntarily participating in this study and signing the informed consent form.Exclusion criteria: (1) Patients with severe complications or severe underlying diseases, such as diabetes, chronic obstructive pulmonary disease, coronary heart disease, severe liver and kidney diseases, etc.; (2) Patients with malignant tumors; (3) Patients with mental diseases.

To construct a structural equation model and obtain reliable and meaningful parameter estimates, most researchers believe that the sample size should be at least 200 (18). Ni Ping et al. (19) pointed out that the sample size for studies on influencing factors of variables should be at least 5-10 times the number of variables. This study includes 14 variables, as well as 10 dimensions from 3 scales (quality of life, social support, and resilience), involving a total of 24 variables. Considering 10% invalid questionnaires, the sample size should be between 134 and 267. Finally, 207 patients were included in this study.

### 2.2 Study methods

#### 2.2.1 Research tools

(1) General information questionnaire: A self-designed questionnaire including age, BMI, marital status, education level, place of residence, etc.

(2) CD-RICS: The CD-RICS was used to assess patients’ resilience level. The scale was developed by Connor-Davidson (20), and localized and revised by Chinese scholars Zhang Jianxin and Yu Xiaonan in 2007 (21), with good reliability and validity (Cronbach’s α coefficient = 0.924; split-half reliability = 0.80). The scale includes 3 dimensions and 25 items: tenacity dimension (items 11-23), self-reliance dimension (items 1, 5, 7, 8, 9, 10, 24, 25), and optimism dimension (items 2, 3, 4, 6). A Likert 5-point scoring method was adopted, ranging from “very inconsistent” to “very consistent”, scored 0-4 points respectively. The total score ranges from 0 to 100 points, with higher scores indicating higher resilience levels.

(3) Social Support Rating Scale: The Social Support Scale (22) was used to assess patients’ social support level. The scale was compiled by Xiao Shuiyuan in 1986, with 10 items, including 3 dimensions: objective support, subjective support, and utilization of social support, to measure the degree of social support received by individuals. The total score is 66 points, with 12 points as the minimum score. Higher scores indicate better social support. The scale has good reliability and validity (Cronbach’s α coefficient = 0.920).

(4) Quality of Life Scale for IBD Patients: The Inflammatory Bowel Disease Quality of Life Questionnaire was used to assess patients’ quality of life. The scale involves 4 dimensions: intestinal symptoms, systemic symptoms, emotional function, and social function, with a total of 32 items. Each item is evaluated by a Likert 7-point scoring method, with scores ranging from 1 to 7. The total score ranges from 32 to 224 points, with higher scores indicating better quality of life. The scale has good reliability and validity (Cronbach’s α coefficient = 0.95, split-half reliability = 0.90), and has been widely used in the evaluation of quality of life in IBD patients (23).

#### 2.2.2 Survey methods

Under the guidance of a professional clinician, two nursing graduate students who underwent standardized training conducted the questionnaire survey. Paper questionnaires were distributed in the outpatient department of gastrointestinal surgery. Before filling out the questionnaires, patients were informed of the purpose, significance, and requirements of the survey, and signed the informed consent form. All questionnaires were distributed and collected on the spot. If patients have questions about the questionnaire during completion, a professional clinician will provide detailed explanations until the patient fully understands. After completion, researchers checked the questionnaires, supplemented any omissions on the spot, and eliminated questionnaires with regular answers.

#### 2.2.3 Statistical processing

After double-entry and verification of data, statistical analysis was performed using SPSS 26.0 and AMOS 24.0 software, with a test level of α = 0.05.

① The general information of IBD patients was statistically described using the number of cases and composition ratio;
② The scores of quality of life, social support, resilience, and their dimensions were described using mean ± standard deviation( ± s);
③ Pearson correlation analysis was used to analyze the correlation among quality of life, social support, and resilience;
④ A structural equation model was constructed to analyze and verify the relationship and action path among quality of life, social support, and resilience, and the mediating effect of the model was tested by the bias-corrected percentile Bootstrap method.

## 3 Results

A total of 207 questionnaires were distributed in this study, and 207 valid questionnaires were recovered, with an effective recovery rate of 100%.

### 3.1 General information of IBD patients

Among the 207 IBD patients, 116 were male (56.04%) and 91 were female (43.96%); the age was (44.24 ± 14.10) years; 32 cases (15.46%) were unmarried, 170 cases (82.13%) were married, and 5 cases (2.42%) were divorced or widowed; 112 cases (54.11%) had ulcerative colitis, while 95 cases (45.89%) had Crohn’s disease.118 cases (57.00%) were in remission, 44 cases (21.26%) were mild, 38 cases (18.36%) were moderate, and 7 cases (3.28%) were severe. See **Table 1** for details.

**Table 1:** General information of IBD patients (n=207).

General information	Group	Number of items(*n*)	Composition ratio(%)
Gender	Men	116	56.04
	Women	91	43.96
level of education	Junior high level and below	76	36.71
	High School/Vocational School	25	12.08
	College degree or above	106	51.21
Marital status	Unmarried	32	15.46
	Married	170	82.13
	Divorce/Widowhood	5	2.42
Living environment	Town	152	73.43
	Rural	55	26.57
Disease type	Ulcerative colitis	112	54.11
	Crohn’s disease	95	45.89
Duration of illness	≤5 years	124	59.90
	5~10 years	44	21.26
	≥10 years	39	18.84
Severity of disease	Remission period	118	57.00
	Mild	44	21.26
	Moderate	38	18.36
	Severe	7	3.28
Medication therapy	Yes	198	95.65
	No	9	4.35
Surgical treatment	Yes	24	11.59
	No	183	88.41

### 3.2 Current status of resilience, social support, and quality of Life in IBD patients

The results of this study showed that the total resilience score of IBD patients was (61.58 ± 22.37) points; the total social support score was (43.37 ± 11.46) points; the total quality of life score was (182.22 ± 31.94) points. See **Table 2** for details.

**Table 2:** Scores of resilience, social support, and quality of life in 207 IBD patients (points, ± s).

Item	Number of items	Score
Resilience	25	61.58 ± 22.37
Tenacity	13	31.23 ± 12.57
Optimism	4	10.13 ± 4.07
Strength	8	20.22 ± 7.31
Social Support	10	43.37 ± 11.46
Objective Support	3	12.77 ± 4.99
Subjective Support	4	23.18 ± 6.06
Utilization of Social Support	3	7.42 ± 2.52
Quality of Life	32	182.22 ± 31.94
Intestinal Symptoms	10	59.30 ± 9.94
Systemic Symptoms	5	26.66 ± 5.67
Emotional Function	12	66.97 ± 13.23
Social Function	5	29.30 ± 5.79

### 3.3 Correlation analysis among resilience, social support, and quality of life in IBD patients

The total resilience score and its dimensions were positively correlated with the total social support score and its dimensions (P<0.01); the total resilience score and its dimensions were positively correlated with the total quality of life score and its dimensions (P<0.01); the total social support score and its dimensions were also positively correlated with the total quality of life score and its dimensions (P<0.01). Additionally, all dimensions of resilience were positively correlated among patients with inflammatory bowel disease (P < 0.01), all dimensions of social support were positively correlated (P < 0.01), and all dimensions of quality of life were positively correlated (P < 0.01). See **Table 3** for details.

**Table 3:** Correlation analysis among resilience, social support, and quality of life in IBD patients.

Item	Optimism	Strength	Tenacity	Total resilience score	Subjective support	Objective support	Utilization of support	Total social support score	Intestinal symptom	Systemic symptom	Emotional ability	Social capacity	Total quality of life score
Optimism	1	–	–	–	–	–	–	–	–	–	–	–	–
Strength	0.817^**^	1	–	–	–	–	–	–	–	–	–	–	–
Tenacity	0.743^**^	0.807^**^	1	–	–	–	–	–	–	–	–	–	–
Total Resilience Score	0.866^**^	0.928^**^	0.960^**^	1	–	–	–	–	–	–	–	–	–
Subjective Support	0.406^**^	0.411^**^	0.390^**^	0.427^**^	1	–	–	–	–	–	–	–	–
Objective Support	0.325^**^	0.370^**^	0.359^**^	0.382^**^	0.578^**^	1	–	–	–	–	–	–	–
Utilization of Support	0.498^**^	0.523^**^	0.475^**^	0.528^**^	0.458^**^	0.575^**^	1	–	–	–	–	–	–
Total Social Support Score	0.466^**^	0.494^**^	0.467^**^	0.508^**^	0.881^**^	0.867^**^	0.712^**^	1	–	–	–	–	–
Intestinal Symptom	0.466^**^	0.507^**^	0.401^**^	0.476^**^	0.375^**^	0.247^**^	0.306^**^	0.373^**^	1	–	–	–	–
Systemic Symptom	0.469^**^	0.545^**^	0.458^**^	0.520^**^	0.414^**^	0.368^**^	0.447^**^	0.477^**^	0.750^**^	1	–	–	–
Emotional Ability	0.492^**^	0.558^**^	0.447^**^	0.522^**^	0.447^**^	0.353^**^	0.430^**^	0.485^**^	0.812^**^	0.847^**^	1	–	–
Social capacity	0.439^**^	0.444^**^	0.380^**^	0.438^**^	0.337^**^	0.214^**^	0.274^**^	0.332^**^	0.788^**^	0.720^**^	0.765^**^	1	–
Total Quality of Life Score	0.512^**^	0.566^**^	0.460^**^	0.536^**^	0.437^**^	0.327^**^	0.402^**^	0.462^**^	0.924^**^	0.893^**^	0.956^**^	0.871^**^	1

**P<0.01 and *P* < 0.01 indicates statistical significance; -, blank entry.

### 3.4 Mediating effect of social support between quality of life and resilience

To further explore the relationship among quality of life, social support, and resilience in IBD patients, and verify the mediating role of social support between quality of life and resilience, this study established a hypothetical model using the Bootstrap method, with resilience as the independent variable, quality of life as the dependent variable, and social support as the mediating variable between quality of life and resilience, for parameter estimation. The maximum likelihood ratio method was used for model modification and fitting to verify the model.

**Figure 1:**
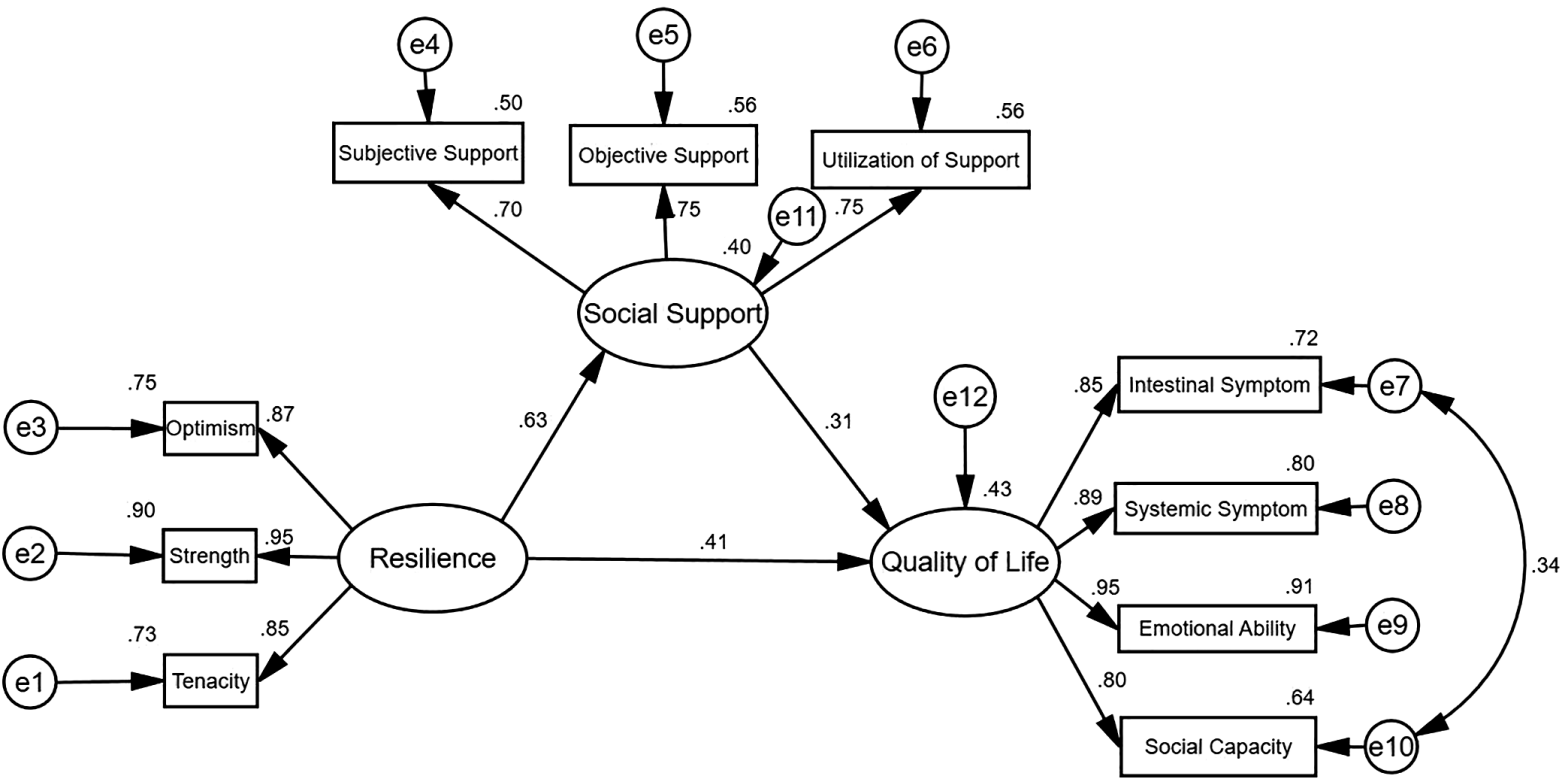
Path relationship model of resilience, social support, and quality of life in IBD patients.

The results of this study showed that χ²/df = 1.628, root mean square error of approximation (RMSEA) = 0.055, Tucker-Lewis index (TLI) = 0.981, comparative fit index (CFI) = 0.987, goodness of fit index (GFI) = 0.955, adjusted goodness of fit index (AGFI) = 0.921. According to the model fitting index standards: χ²/df<3, RMSEA<0.080, TLI>0.900, CFI>0.900, GFI>0.900, AGFI>0.900, the model path fitting was good, and all path coefficients of the model reached a significant level. The fitting model of the mediating effect of social support in IBD patients is shown in **Figure 1**. In addition, the direct effect value of resilience on quality of life was 0.412, with 95%CI (0.282, 0.567), and the result did not include 0, indicating that the direct effect was significant. The indirect effect value of quality of life on resilience was 0.197, with 95%CI (0.070-0.318), and the result did not include 0, indicating that the mediating effect of social support was significant, playing a partial mediating role. The total effect value was 0.609, with 95%CI(0.541~0.683). The mediating effect accounted for 32.35% of the total effect, while the direct effect accounted for 67.65% of the total effect. See **Table 4**.

**Table 4:** Test of mediating effect of social support between resilience and quality of life in IBD patients.

Item	Standardized effect	Boot *SE*	Boot 95%*CI*		*P*	Effect ratio(%)
			LL	UL		
Direct Effect	0.412	0.089	0.282	0.567	<0.001	67.65
Indirect Effect	0.197	0.068	0.070	0.318	<0.001	32.35
Total Effect	0.609	0.043	0.541	0.683	<0.001	

*P* < 0.01 indicates statistical significance.

## 4 Discussion

Analysis of the current status of resilience, social support, and quality of life in IBD patients. The results of this study showed that the total resilience score of IBD patients was (61.58 ± 22.37) points, which was at a moderate level, but lower than the research results of Dong Zhengchuan et al. (24), which may be related to differences in the chronic and recurrent nature of the disease and the long-term physical and mental burden caused by it among different individuals. However, in this study, the total social support score of IBD patients was (43.37 ± 11.46) points, which was higher than the research results of Dong Zhengchuan et al. (25), which may be related to differences in the range of social activity restrictions or stigma caused by the disease in different patients. In terms of quality of life, the total score of IBD patients was (182.22 ± 31.94) points, which was higher than the research results of Zhao Baoning et al. (26), possibly due to differences in sample size. It is worth noting that patients with higher resilience also had higher social support and higher quality of life scores; conversely, those with lower social support had poorer quality of life. The above suggests that there is a certain relationship among resilience, social support, and quality of life in IBD patients.

The direct effect of resilience on the quality of life of IBD patients. This study verified that resilience has a direct positive impact on quality of life, that is, patients with stronger resilience have better quality of life. This result is consistent with the research results of Zhang J et al. (27) and Sehgal P et al. (28): patients with high resilience have higher adaptability, so their quality of life is better. The stress coping theory holds that individuals will adopt active coping strategies or negative escape strategies when facing stress (29). Studies have shown that individuals with high resilience are more likely to adopt active coping strategies, and active coping can improve patients’ ability to manage diseases to a certain extent, reduce psychological distress caused by diseases, and thus improve quality of life (30). In addition, resilience may also indirectly improve physiological functions and social participation by enhancing patients’ disease adaptability, such as compliance with treatment plans and initiative in symptom monitoring, thereby improving quality of life. The above suggests that in future clinical interventions, medical workers should not only provide traditional drug treatment for IBD patients but also pay attention to the cultivation of their psychological resilience, such as enhancing their psychological adaptability through cognitive behavioral therapy or mindfulness training to improve their resilience and further enhance their quality of life.

Resilience indirectly affects the quality of life of IBD patients through social support. The results of this study showed that social support plays a partial mediating role between resilience and quality of life; this indicates that the stronger the resilience of IBD patients, the more actively they will seek and effectively use social support, and the less negative impact on their quality of life. This result is consistent with the findings of Moisoglou I et al. (31) and Yu S et al. (32). This also aligns with existing theories and research: on one hand, Rossi et al. (33) indicate that resilience can alleviate anxiety and depressive symptoms by reducing stress; on the other hand, the mechanisms underlying social support have been extensively elucidated. For instance, the stress buffering hypothesis proposed by Cohen S et al. (34) suggests that social support exerts its effects by mitigating the negative impact of stress on health. Furthermore, Bandura’s social cognitive theory emphasizes the interaction between individual cognition (such as resilience) and environmental factors (such as social support) (35) Integrating Amin SM et al.’s research (36), social support can enhance quality of life by regulating mental health. Thus, resilience as a psychological adaptive capacity may enhance patients’ quality of life by increasing their perception and utilization of social support. Specifically, individuals with high resilience are more likely to actively seek emotional support and instrumental help, and social support can improve quality of life by alleviating disease stress and promoting treatment compliance (37, 38). However, it is worth noting that some patients, despite having high resilience, may still have a low quality of life due to weak social support networks (such as living alone or lacking understanding from relatives and friends). Therefore, in daily clinical practice, medical workers need to pay more attention to patients’ social support systems, in order to build a more perfect support environment for patients through multidisciplinary collaboration (such as patient support groups), to maximize the positive role of resilience.

## 5 Conclusion

In conclusion, the quality of life of IBD patients is at a moderate to high level. Resilience can not only directly affect patients’ quality of life but also indirectly affect it through social support. The results of this study suggest that medical staff should take certain intervention measures to improve the social support of IBD patients, thereby enhancing their quality of life and helping them return to society smoothly.

## 6 Limitations and strengths

This study confirms the mediating role of social support in the relationship between resilience and quality of life among patients with IBD, offering a new perspective and corresponding scientific rationale for improving their quality of life. However, This study also has certain limitations: it only included IBD patients from one hospital, resulting in insufficient sample representativeness. As a cross-sectional study, the structural equation model did not adjust for covariates, potentially introducing bias in the results. Furthermore, it is particularly important to note that psychosocial intervention measures for IBD patients should be validated through longitudinal or interventional studies before clinical application.

## Data Availability

The original contributions presented in the study are included in the article/**Supplementary Material**. Further inquiries can be directed to the corresponding author.
