# Supplementary material for: Study on the correlation between resilience, social support and quality of life in patients with inflammatory bowel disease
Source: Front Psychiatry. 2026 Jan 22;16:1694513. doi: 10.3389/fpsyt.2025.1694513 (PMC12873305; doi:10.3389/fpsyt.2025.1694513)
Supplement: Supplementary file 1 [file Table1.doc]

Table 1 Scores of Resilience, Social Support, and Quality of Life in 207 IBD Patients (points,
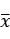
±s )

| Item | Number of Items | Score |
| --- | --- | --- |
| Resilience | 25 | 61.58±22.37 |
| Tenacity | 13 | 31.23±12.57 |
| Optimism | 4 | 10.13±4.07 |
| Strength | 8 | 20.22±7.31 |
| Social Support | 10 | 43.37±11.46 |
| Objective Support | 3 | 12.77±4.99 |
| Subjective Support | 4 | 23.18±6.06 |
| Utilization of Social Support | 3 | 7.42±2.52 |
| Quality of Life | 32 | 182.22±31.94 |
| Intestinal Symptoms | 10 | 59.30±9.94 |
| Systemic Symptoms | 5 | 26.66±5.67 |
| Emotional Function | 12 | 66.97±13.23 |
| Social Function | 5 | 29.30±5.79 |

Table 2 Correlation Analysis among Resilience, Social Support, and Quality of Life in IBD Patients

| Item | Optimism | Strength | Tenacity | Total Resilience Score | Subjective Support | Objective Support | Utilization of Support | Total Social Support Score | Intestinal Symptom | Systemic Symptom | Emotional Ability | Social Capacity | Total Quality of Life Score |
| --- | --- | --- | --- | --- | --- | --- | --- | --- | --- | --- | --- | --- | --- |
| Optimism | 1 | - | - | - | - | - | - | - | - | - | - | - | - |
| Strength | 0.817 | 1 | - | - | - | - | - | - | - | - | - | - | - |
| Tenacity | 0.743 | 0.807 | 1 | - | - | - | - | - | - | - | - | - | - |
| Total Resilience Score | 0.866 | 0.928 | 0.960 | 1 | - | - | - | - | - | - | - | - | - |
| Subjective Support | 0.406 | 0.411 | 0.390 | 0.427 | 1 | - | - | - | - | - | - | - | - |
| Objective Support | 0.325 | 0.370 | 0.359 | 0.382 | 0.578 | 1 | - | - | - | - | - | - | - |
| Utilization of Support | 0.498 | 0.523 | 0.475 | 0.528 | 0.458 | 0.575 | 1 | - | - | - | - | - | - |
| Total Social Support Score | 0.466 | 0.494 | 0.467 | 0.508 | 0.881 | 0.867 | 0.712 | 1 | - | - | - | - | - |
| Intestinal Symptom | 0.466 | 0.507 | 0.401 | 0.476 | 0.375 | 0.247 | 0.306 | 0.373 | 1 | - | - | - | - |
| Systemic Symptom | 0.469 | 0.545 | 0.458 | 0.520 | 0.414 | 0.368 | 0.447 | 0.477 | 0.750 | 1 | - | - | - |
| Emotional Ability | 0.492 | 0.558 | 0.447 | 0.522 | 0.447 | 0.353 | 0.430 | 0.485 | 0.812 | 0.847 | 1 | - | - |
| Social capacity | 0.439 | 0.444 | 0.380 | 0.438 | 0.337 | 0.214 | 0.274 | 0.332 | 0.788 | 0.720 | 0.765 | 1 | - |
| Total Quality of Life Score | 0.512 | 0.566 | 0.460 | 0.536 | 0.437 | 0.327 | 0.402 | 0.462 | 0.924 | 0.893 | 0.956 | 0.871 | 1 |

Note: P＜0.01

Table 3 Test of Mediating Effect of Social Support between Resilience and Quality of Life in IBD Patients

| Item | Standardized Effect | Boot *SE* | Boot 95%*CI* | | *P* | Effect Ratio（%） |
| --- | --- | --- | --- | --- | --- | --- |
|  |  |  | LL | UL |  |  |
| Direct Effect | 0.412 | 0.089 | 0.282 | 0.567 | ＜0.001 | 67.65 |
| Indirect Effect | 0.197 | 0.068 | 0.070 | 0.318 | ＜0.001 | 32.35 |
| Total Effect | 0.609 | 0.043 | 0.541 | 0.683 | ＜0.001 |  |
